# The effect of glucosamine sulphate on osteoarthritis: design of a long-term randomised clinical trial [ISRCTN54513166]

**DOI:** 10.1186/1471-2474-6-20

**Published:** 2005-04-26

**Authors:** Rianne M Rozendaal, Bart W Koes, Harrie Weinans, Elian J Uitterlinden, Gerjo JVM van Osch, Abida Z Ginai, Jan AN Verhaar, Sita MA Bierma-Zeinstra

**Affiliations:** 1 Department of General Practice, Erasmus MC, University Medical Centre Rotterdam, the Netherlands; 2 Department of Orthopaedics, Erasmus MC, University Medical Centre Rotterdam, the Netherlands; 3 Department of Radiology, Erasmus MC, University Medical Centre Rotterdam, the Netherlands

## Abstract

**Background:**

Pharmacological treatment for osteoarthritis (OA) can be divided into two groups: symptom-modifying drugs and disease-modifying drugs. Symptom-modifying drugs are currently the prescription of choice for patients with OA, as disease-modifying drugs are not yet available in usual care. However, there has recently been a lot of debate about glucosamine sulphate (GS), a biological agent that is thought to have both symptom-modifying and disease-modifying properties. This assumption has yet to be proved.

The objective of this article is to present the design of a blind randomised clinical trial that examines the long-term symptom-modifying and disease-modifying effectiveness of GS in patients with hip OA. This trial is ongoing and will finish in March 2006.

**Methods/design:**

Patients with hip OA meeting the ACR-criteria are randomly allocated to either 1500 mg of oral GS or placebo for the duration of two years. The primary outcome measures, which are joint space narrowing (JSN), and change in the pain and function score of the Western Ontario McMaster Universities Osteoarthritis index (WOMAC), are determined at baseline and after two years of follow-up during the final assessment. Intermediate measures at three-month intervals throughout the trial are used to study secondary outcome measures. Secondary outcome measures are changes in WOMAC stiffness score, quality of life, medical consumption, side effects and differences in biomarker CTX-II.

## Background

Pharmacological treatment for osteoarthritis (OA) can be divided into two groups: symptom-modifying drugs and disease-modifying drugs [1]. Symptom-modifying drugs are at present the prescription of choice for patients with OA. Drugs in this group are: simple analgesics (such as acetaminophen) and non-steroidal anti-inflammatory drugs (NSAIDs). Both acetaminophen and NSAIDs are effective in relieving symptoms of OA. In more severe stages of the disease NSAIDs are more effective, however, they are also the cause of serious side effects [2].

Disease-modifying drugs (i.e. drugs which alter disease progression) are not yet available in usual care. Although there has recently been a lot of debate about some biological agents that are thought to have both symptom-modifying and disease-modifying properties, results from previous trials have not been convincing. Of these biological agents, glucosamine sulphate seems to be the most promising.

Glucosamine sulphate (GS) has been shown to be an effective symptom-modifying agent, with effect sizes ranging from moderate to high [3,4]. In four trials that compared GS and NSAIDs, GS was found to be as effective as, or slightly more effective than NSAIDs [4]. Together with the fact that no serious adverse events have been reported concerning GS [3,4] this implies that GS may be a good alternative to NSAIDs. However, due to publication bias and due to quality issues in the trials studying GS, it may well be that reported effect sizes are exaggerated. A more recent trial studying the effect of GS did not find a difference between GS and placebo [5]. Also, several other uncertainties exist concerning the symptom-modifying properties of GS. For example, most trials were only of short-term duration (e.g. mean 6.25 weeks [4]) and it is therefore not possible to draw conclusions about the long-term efficacy. Another problem is that the mechanism behind the improvement of symptoms due to GS is not known. If GS directly influences the remaining cartilage, it would seem plausible that the symptomatic effect is greater in people with mild to moderate OA than in people with more severe OA, because there is more cartilage remaining in the first group. However, this possible difference in effect between different stages of OA has not been tested yet in a randomised clinical trial (RCT). These uncertainties make further study into the magnitude of long-term symptom-modifying effects in different stages of the disease justified.

Concerning disease-modifying effects, two recent long-term (three years) trials in patients with knee OA did report some evidence that GS affected the progression of OA [6,7]. Progressive joint space narrowing in the narrowest medial compartment of the tibiofemoral joint was used to define progression of knee OA (as recommended by a task force of the OA research society [8]). Whereas joint space narrowing (JSN), had significantly progressed in the placebo groups, it had not in the groups that were taking GS. This implies that daily intake of GS acted against progression of OA. However, these results are controversial, because both trials lacked appropriate and standardised protocols for taking radiographs. Although it is not likely that this influenced the results much [9], it is necessary to reproduce them in a study with well-standardised protocols. These two long-term trials both looked at the effect of GS on knee OA, no trial has been or is being performed yet that looks at its effect on hip OA.

Based on the above, we designed a long-term trial to answer our main question: Does glucosamine sulphate favourably modify progression of osteoarthritis? Because there still is uncertainty about the symptom-modifying properties of GS we will also try to answer three secondary questions: Does GS have the same effects in all stages of OA? What is the long-term cost-effectiveness of addition of GS to usual care? And, does GS prevent the onset or progression of OA in the contralateral hip joint? Additional to these clinical questions, the data will be used to look at changes on cell-level caused by GS to learn more about its possible mechanisms of action. All results derived from this trial will be published using our International Standardised Randomised Controlled Trial Number (ISRCTN).

In this article we will present the detailed protocol of the trial. This trial is ongoing; at the moment all patients are included and have passed the first 9 months of the follow-up period.

## Methods/design

### Study design

This study is a randomised, blinded, placebo-controlled trial. All actors in this trial, who may cause bias, are blinded to treatment allocation: the patient, who is the assessor of the symptomatic outcomes, the researcher, who is the assessor of the objective outcomes, and the caregiver. The analyses will also be performed blind. The study design was approved by the Medical Ethics Committee at the Erasmus MC – university medical centre Rotterdam. All patients gave written informed consent.

### Patient selection

General practitioners in the Rotterdam area agreed to search their electronic medical record for patients diagnosed with hip OA and for patients with symptoms associated with hip OA (i.e. persistent hip pain in combination with NSAID use). These patients are contacted by their general practitioner and informed about the trial. For more information, patients can forward their contact details to the researchers. These patients then receive an extensive information folder containing all the information needed to make an informed decision about participation in the study. This folder has been reviewed and approved by the medical ethics committee.

The information folder also contains an informed consent form, which patients need to fill out if they want to participate in the study. Patients who give written informed consent are contacted by phone for a preliminary check of the inclusion and exclusion criteria. People meeting these criteria are invited to the research centre of Erasmus MC for a baseline-measurement, during which the criteria can be checked more precisely.

### In- and exclusion criteria

Patients are eligible for inclusion when they meet one of the ACR criteria for hip OA [10]. Patients that have already undergone hip replacement surgery or those on the waiting list for joint replacement are not included in the study. Neither eligible patients with a Kellgren & Lawrence (K-L) score of 4 [11], nor people with renal and/or hepatic disease, diabetes mellitus or a disabling co-morbidity are included. Finally, patients unable to understand Dutch questionnaires are excluded from participation.

### Sample size

The sample size was calculated primarily to detect clinically relevant differences in radiological progression of the affected joints between the two groups (treatment and placebo) after two years of follow up. To detect a difference of 0.25 mm in radiological progression (SD 0.5) between the intervention and placebo groups (power 80%, alpha 5%, one-tailed testing) after two years of follow-up, 63 patients with hip osteoarthritis are needed per group. These calculations are based on an average change of 0.33 mm in joint space (SD 0.5) during one year of follow-up of patients with hip osteoarthritis [12].

Fewer patients are needed to detect relevant clinical differences: to detect a difference of 25% in pain (Western Ontario McMaster Universities Osteoarthritis index (WOMAC)) with one-tailed testing, a power of 80%, and alpha 5% (Mean 4.83, SD 2.25 [13]) 55 patients per group are needed. To detect the same difference in function (WOMAC) (mean 4.81, SD 2.18 [13]) 51 patients are needed per group.

As we expect a 20% loss to follow-up, we need to include 150 patients. However, to create options for studying effect-modification by type and severity of osteoarthritis, we oversized this trial to 220 patients (110 in each group).

### Intervention

Patients who participate in the trial are randomised to either GS or a placebo for the duration of two years. To ensure a daily intake of 1500 mg GS, they are required to take two pills each day. The GS and placebo pills are identical in taste and appearance and were delivered in identical plastic bottles. This will ensure true blinding of the patients and of the researchers. Blinding of the patients will be tested after two years; if people can guess what sort of pills they were taking, this might have an influence on the subjective measures. This will therefore be taken into account in the analysis of the data.

The Department of Nutritional Sciences at Numico Research BV manufactured the pills used in this trial.

### Randomisation

Following informed consent and baseline assessments, patients are allocated to the intervention or control group using a blinded randomisation list. The randomisation list contains four different strata and is randomised per block of six numbers. This list was generated with a computer by an independent researcher. This researcher also handled labelling the pill-bottles with the randomisation numbers. The researchers involved in this project received all bottles after they were labelled, ensuring blinding to treatment allocation. The randomisation list with the key to treatment allocation will be kept in a safe until the end of the trial. To be able to perform the analyses blinded, the allocation to treatment A and treatment B will be provided, but not the key to A and B.

People are assigned to one of the four different strata on the basis of the Kellgren-Lawrence score of the hips, knees and hands. A researcher (RMR) will score all the radiographs according to the Kellgren-Lawrence score. The outline of the four different strata is given in table 1. Once the correct stratum is established at baseline, the patient is given the subsequent unique four-digit randomisation number from his/her stratum on the randomisation list. This number is used for labelling study materials and data. By stratifying, patients are optimally distributed to GS and placebo in the different strata, which makes comparing people with mild OA to people with moderate-severe OA, and comparing people with local OA to people with generalised OA possible. In this way, we will be able to study whether effect of treatment depends on severity or localisation of OA.

**Table 1:** Outline of the randomisation strata

	**Hip radiograph**	**Knee and hand radiographs**	**Type**
**Group 1**	K-L score < 2	K-L score < 2 for hands and knees	mild + localised
**Group 2**	K-L score < 2	K-L score ≥ 2 for hands and/or knees	mild + generalised
**Group 3**	K-L score ≥ 2	K-L score < 2 for hands and knees	moderate/severe + localised
**Group 4**	K-L score ≥ 2	K-L score ≥ 2 for hands and/or knees	moderate/severe + generalised

### Measurements

Data for the primary and secondary outcome measures are being collected at different time-points throughout the trial. An overview of the timing of the measurements and the outline of the primary and secondary outcome measures is given in table 2.

**Table 2:** Timing of measurements and outline of primary and secondary outcome measures

	**0 m** B.A.	**3 m** Q	**6 m** Visit	**9 m** Q	**12 m** Visit	**15 m** Q	**18 m** Visit	**21 m** Q	**24 m** F.A.
**Primary outcome measures**									
Joint space width	**x**								**x**
Pain score (WOMAC)	**x**								**x**
Function score (WOMAC)	**x**								**x**
									
**Secondary outcome measures**									
Subchondral bone quality	**x**								**x**
Stiffness score (WOMAC)	**x**	**x**	**x**	**x**	**x**	**x**	**x**	**x**	**x**
Quality of life (EuroQol EQ-5D)	**x**	**x**	**x**	**x**	**x**	**x**	**x**	**x**	**x**
Medical consumption	**x**	**x**	**x**	**x**	**x**	**x**	**x**	**x**	**x**
Side effects		**x**	**x**	**x**	**x**	**x**	**x**	**x**	**x**
CTX-II	**x**		**x**		**x**		**x**		**x**
									
**Possible confounders/Effect modifiers**									
Type of OA (localised – generalised)	**x**								
Radiological severity	**x**								**x**
Joint function	**x**								**x**
Age	**x**								
Gender	**x**								
Activity level	**x**	**x**	**x**	**x**	**x**	**x**	**x**	**x**	**x**
Co-interventions	**x**	**x**	**x**	**x**	**x**	**x**	**x**	**x**	**x**
Compliance (BMQ)		**x**	**x**	**x**	**x**	**x**	**x**	**x**	**x**
Compliance (pill count)			**x**		**x**		**x**		**x**

Note: 0 m: 0 month of follow up, 3 m: 3 months of follow up etc. B.A.: baseline assessment. Visit: 6 monthly visit. F.A.: final assessment

In brief, the trial starts for every patient with a baseline assessment at the research centre. At the end of this assessment, patients receive a supply of GS or placebo sufficient for seven months. After the baseline assessment, patients will receive a questionnaire every three months, which has to be returned to the researchers, except from those at 6, 12 and 18 months after baseline, which will be collected by the researchers during a home visit. After two years, patients return to the research centre for the final assessment, which marks the end of the trial. The collection of the outcome measures is described in the following sections.

#### Radiographs

Radiographs are taken during the baseline assessment and during the final assessment two years later.

At baseline, three anteroposterior (AP) radiographs are taken, one of the pelvis, one of both knees, and one of both hands. All radiographs are used to establish what stratum the subject belongs to (table 1). The radiographs of hands and knees will not be used to determine outcome measures and will therefore not be repeated at follow up. As follows from table 1, people with knee and/or hand OA are stratified to one of the 'generalised OA' groups (2 or 4).

A highly standardised protocol is used to make the weight-bearing, AP pelvic radiographs at baseline and follow-up, allowing for a correct measurement of our primary outcome variable: joint space narrowing. The patients' feet are positioned alongside a frame, which was designed to ensure 15° internal rotation of the hips. A second frame (no internal rotation) is available for patients with severe mobility restrictions of the hips. The frame used during the baseline radiograph of a patient will also be used two years later for his/her follow up radiograph. Patients are asked to stand upright. If present, flexion in hips or knees is recorded. Protocol for the pelvic X-ray further states that focus-to-film distance should be 130 cm and that the X-ray beam should be centred on the superior aspect of the pubic symphysis. The X-rays are digitised.

The X-rays from baseline and final assessment will be analysed side by side. The minimal joint space width (JSW) will be identified from the baseline X-ray by assessing four different points: medial, axial, superior and lateral [14]). The researcher will also identify a point that appears to be the minimal JSW. From these five points the actual minimal JSW will be determined. This point will be used to measure changes in joint space width over the two-year follow up period.

#### Dexa-scan

During the baseline and final assessment, a Dual Energy X-ray Absorptiometry (DEXA) scan will be used to make scans of the pelvis. A frame similar to the one used for the radiograph of the hips is also used to make the DEXA-scan, ensuring 15° internal rotation of the hips. The scan will be used to study quantitative changes in subchondral bone density both of the affected joint and of the contra-lateral joint. Subchondral bone-density alterations might indicate osteoarthritic progression. This long-term trial can be helpful to determine whether pre-clinical OA can be recognised from a DEXA-scan. And, if so, whether GS prevents the onset or progression of OA in the pre-clinical stage.

#### Physical examination

A physical examination is carried out at baseline and is repeated during the final assessment. At baseline, this test is first of all used to check part of the inclusion criteria. Various tests are also carried out to check for co-existing musculoskeletal disorders. Findings from the physical examination will be used as baseline characteristics, and to register clinical signs and joint function after two years of follow up. Joint function is established by assessing pain due to joint motion, and by measuring limitation of joint motion with a two-arm goniometer.

#### Questionnaires

Throughout the study, patients will fill out a total of nine questionnaires. The first during the baseline assessment, followed by a questionnaire every three months in the following two years (including the last one during the final assessment).

The baseline questionnaire is used to measure different patient characteristics (age, gender, race, social status, Body Mass Index (BMI)), disease related characteristics (localisation of symptoms, duration of symptoms, family history) and co-morbidities. Of these characteristics, BMI and co-morbidities will be monitored throughout the trial.

Three validated instruments are used in all nine questionnaires: the WOMAC questionnaire will be used to establish severity of clinical status. It contains subscales for pain, stiffness and function. The WOMAC questionnaire is extensively validated and recommended for clinical assessment in osteoarthritis trials by the WHO [15]. The EuroQol (EQ-5D) will be used to measure quality of life, because of the usefulness of this scale in cost-effectiveness analysis [16,17]. The cost-effectiveness analysis will also be based on employment status, sick leave, changes in work-tasks or other work-related adjustments, and on medical consumption. The SQUASH questionnaire is used to measure load level in work and sports [18].

In the eight follow-up questionnaires, patients will be asked to answer questions about alterations in their symptoms (i.e. whether they improved or deteriorated), which will be measured with a 7-point Likert scale. Also, compliance to treatment is measured with the Brief Medication Questionnaire (BMQ) [19].

#### Laboratory assessments

At baseline, two samples of blood are collected. The first to measure the erythrocyte sedimentation rate (ESR), which is used for the inclusion criteria (ACR-criteria). The second sample is stored at -20°C to create options for future DNA-research, for which patients gave separate written informed consent.

Throughout the study, we will collect samples of second-morning void urine of all patients. In urine a marker of cartilage degradation can be found, called CTX-II. In the Rotterdam study [20] this marker was found to be predictive of radiological progression of hip and knee OA. It may therefore be used to assess the effect of treatment on the progression of OA. Urine samples will be collected at baseline and once every six months during follow up. At the end of the study a total of five samples will be available from every patient. These urine samples are stored at -80°C. If promising new markers are discovered during the course of the study, these can also be included in the analysis.

### Half-yearly visits

Every six months one of the researchers will visit the patients at home. The main reason for this visit is to provide the patient with new pills (sufficient for seven months). To be able to calculate compliance to treatment, the pills remaining of the previous supply will be collected. The amount of remaining pills combined with the score on the compliance questionnaire (BMQ) will give a good indication of the actual amount of pills the patient has been taking. Finally, a sample of second-morning void urine on the day of the visit will be collected.

### Analyses

The researchers will be aware of allocation to treatment A or B at the time of the statistical analyses, but will not know which group received GS and which group received the placebo. All analyses will take place after the trial has finished, no intermediate analyses will be performed.

Success of randomisation and normality of outcome measures will be checked before actual analyses are done. Differences in the primary outcome measures JSN and WOMAC (pain and function) between the intervention and placebo group will be analysed on the basis of the 'intention to treat' principle using linear regression models. Additionally a per-protocol analysis will be done. When it turns out that randomisation was (partially) unsuccessful, we will adjust for differences in prognosis. Using baseline characteristics, we can identify factors that influence outcome of the study. Factors that change the outcome with 10% will be regarded as confounders and will therefore be added to the regression-model.

A cost-effectiveness analysis will be performed from a social and a patient perspective, looking at differences in direct and indirect health care cost between the two groups (GS and placebo). If the trial does not show a difference in disease parameters (WOMAC) and quality of life (EuroQol) between the GS and the placebo group, the analysis will be reduced to a cost minimisation analysis. This form of analysis evaluates the efficacy of treatment based solely on direct and indirect costs. If the study does find a positive difference in disease parameter and/or quality of life a cost-effectiveness ratio can be determined with on the one hand the costs and savings and on the other hand the disease-specific parameters and also quality of life.

### Current status

A total of 40 GP's were found willing to participate in the study. They sent a total of 600 letters to inform possible eligible patients of the study. We received 417 requests for additional information and thus sent an equal amount of information folders. Of these 417 people 250 returned a written informed consent. Eventually 222 people entered the study. Of the 28 people that did not enter the study, most did not meet the inclusion criteria and a few people changed their mind and withdrew their informed consent before randomisation.

We started including patients at the end of September 2003 and the last patient was included on March 15^th ^of 2004. This means the study will run until March 2006. The first results will be available around September 2006.

## Competing interests

The Department of Nutritional Sciences of Numico Research BV provided the pills used in this study free of charge. Numico Research BV was not involved in the design of this trial nor will they be involved in any other aspect of the trial.

## Authors' contributions

SMABZ and HW conceived of the study and developed the design of this randomised clinical trial. SMABZ participated in writing the article, HW contributed to its content. BWK, JANV and GJVMvO contributed to the design of the study and the content of the article. EJU and AZG contributed to the content of the article. RMR is conducting the research, participated in the completion of the study design and wrote the article.

## Pre-publication history

The pre-publication history for this paper can be accessed here:


